# Alteration of Cholesterol Sulfate/Seminolipid Ratio in Semen Lipid Profile of Men With Oligoasthenozoospermia

**DOI:** 10.3389/fphys.2019.01344

**Published:** 2019-10-29

**Authors:** Patrizia Lopalco, Rita Vitale, Yoon Sung Cho, Pasquale Totaro, Angela Corcelli, Simona Lobasso

**Affiliations:** ^1^Department of Basic Medical Sciences, Neuroscience, and Sense Organs, University of Bari Aldo Moro, Bari, Italy; ^2^Centre for Medically Assisted Procreation, Santa Maria Hospital, Bari, Italy

**Keywords:** oligoasthenozoospermia, cholesterol sulfate, lipids, MALDI-TOF/MS, sperm

## Abstract

The reduction of sperm motility and count, or oligoasthenozoospermia, is one of the major causes of reduced fertility or infertility in men. Lipid composition of spermatozoa is important in determining their functional characteristics, in particular on motility, acrosomal exocytosis or fusogenic properties of the sperm. Here we investigated the levels of semen lipids in 11 infertile patients with severe oligoasthenozoospermia and 9 normozoospermic subjects with normal motility values. Sperm polar and neutral lipids were analyzed by thin-layer chromatography (TLC) and matrix-assisted laser desorption and ionization time-of-flight mass spectrometry (MALDI-TOF/MS). Semen of patients with oligoasthenozoospermia showed a reduction of the degree of fatty acid unsaturation in the phospholipids chains that might affect the membrane fluidity. Furthermore, a significant higher cholesterol sulfate/seminolipid ratio was found in semen of oligoasthenozoospermic patients than in subjects with normal motility values, suggesting a critical role of sulfolipids in semen quality. The results may facilitate the understanding of the role of lipids on male fertility and offer interesting perspectives to find innovative treatments for oligoasthenozoospermia.

## Introduction

Although the knowledge of the physiology of the human reproduction has advanced to a marked degree thanks to several studies on semen and related problems, the assessment of male infertility is impaired by limited diagnostic and therapeutic tools and its cause is still unknown.

The semen consists of fluid components, i.e., the seminal fluid, in which there are the cellular constituents, i.e., the spermatozoa. The seminal fluid is produced in different proportion by several glands: the prostate, the seminal vesicles and the bulbourethral glands (Mann and Lutwak-Mann, 1981). Spermatozoa, produced by testes, represent only a small portion of the whole semen, from 1 to 5% of the total volume (Mortimer, 1994).

The unusual molecular composition and the architecture of the sperm plasma membrane play an important role in determining the specific sperm properties and functions. Despite some variations between different mammalian species, in general the sperm plasma membrane shares a unique lipid composition: (1) an unusually low content of cholesterol (Chol); (2) an high proportion of plasmalogen, i.e., lipids containing a fatty acid chain attached as a vinyl ether and another acyl chain with an ester linkage; (3) the *sn*-2 position of the glycerol esterified mainly with long-chain polyunsaturated fatty acids (PUFAs); and (4) an high content of sulfogalactolipids (Kornblatt et al., 1972; Mann and Lutwak-Mann, 1981; Aitken, 1999).

Usually, the *sn*-1 position of the glycerol backbone is typically derived from saturated aliphatic chains with a carbon chain length of 16 atoms, while the *sn*-2 position is most commonly occupied by long-chain PUFAs (Evans et al., 1980). Significant levels of PUFAs (predominantly docosahexaenoic acid, 22:6) have been found in the chemical structures of the main phospholipids of sperm membrane (Mann and Lutwak-Mann, 1981; Aitken, 1999; Schiller et al., 2000).

In general, the high presence of the double bonds in the lipids increases chemical reactivity favoring the action of ROS and the processes of lipid peroxidation (Brodnitz, 1968; de Lamirande et al., 1997; Fuchs et al., 2012). However, the level of seminal ROS is restricted by seminal antioxidants and a specialized scavenger system, which have beneficial effects on sperm parameters and defend the sperm membrane against lipoperoxidation (Lenzi, 1996).

As said above, glycolipids represent an important class of lipids present in sperm membrane; generally, glycolipids are formed by the addition of a glycosidic head group to ceramide in the membranes of the vertebrates. The only exception is the glycolipid seminolipid, having a sulfo-galactosyl glycerol in its molecular structure. The seminolipid, also known as sulfogalactosylglycerolipid (SGG), is the major anionic glycolipid found only in sperm plasma membrane of mammals. The importance of the SGG for spermatogenesis, as well as for the acrosome reaction and the fertilization fusion is well-known [see (Vos et al., 1994; Tanphaichitr et al., 2018)]. It has been demonstrated that the SGG is highly concentrated in the apical ridge region of the sperm head plasma membrane in freshly ejaculated boar sperm, whereas it migrates toward the equatorial region of the sperm head plasma membrane during capacitation by Ca^2+^ ions (Flesch and Gadella, 2000). When it is localized in the apical region, the SGG probably prevents the acrosome reaction by stabilizing lamellar lipid bilayer of the plasma membrane (Vos et al., 1994). In mature germ cells and sperm cells it is exclusively present, like other glycolipids, in the outer lipid leaflet of the plasma membrane (Shirley and Schachter, 1980; Gadella et al., 1993). The SGG is in contact with the glycocalyx components by linkage to its seminolipid immobilizing protein (SLIP-1); both the SLIP-1 and free seminolipid play an important role in binding to the zona pellucida of the egg by electrostatic interactions (Tanphaichitr et al., 1998; White et al., 2000).

As regards neutral lipid composition of sperm membranes, there are major variations between different species. Human mature sperm cells contain rather high Chol to phospholipids molar ratio (Keber et al., 2013). The Chol content of sperm membranes generally decreases during epididymal transit in various species, resulting in the decreased Chol to phospholipids molar ratio (Cross, 1998). The content of Chol in the sperm membrane is further decreased in the female reproductive tract, by the process of capacitation leading to the acrosome reaction. It is known that albumin serves as a Chol acceptor during capacitation; thus, albumin is included *in vitro* fertilization in order to get efficient embryo production (Langlais and Roberts, 1985; Iborra et al., 2000).

The cellular sterol content of sperm cells seems to be linked to the length of capacitation (Yanagimachi, 2005): species with high Chol content (such as human and bull) require longer periods for optimal capacitation (about 6–8 h). Whereas boar and ram sperm with lower Chol content require shorter periods (a couple of hours) (Yanagimachi, 2005). Anyway, the loss of Chol is known to increase the fluidity of the sperm membrane, which is an important requisite for the final steps of sperm maturation in the female reproductive tract. It has been demonstrated that sperm membrane of patients with idiopathic infertility displayed a higher Chol to phospholipids ratio (Sugkraroek et al., 1991) and that high Chol levels in human sperm are negatively correlated with semen quality (Zalata et al., 2010).

Besides Chol, minor amounts of desmosterol, cholesteryl esters, and cholesterol sulfate (Chol-S) can be found in sperm membrane (Mann and Lutwak-Mann, 1981; Sion et al., 2001). Functional and structural roles of this unusual sterol sulfates have been demonstrated. Chol-S accumulates in spermatozoa during epididymal transit and it acts as a membrane stabilizer and inhibitor of acrosin (Roberts, 1987).

It is known that sperm motility is directly related to membrane lipid composition, even if the complex molecular mechanisms of asthenozoospermia remain still unclear. Some years ago, a study of metabolic characterization of seminal plasma from patients with asthenozoospermia using a non-targeted metabolomics approach demonstrated significant changes in lipids metabolism, choline metabolism, Chol metabolism (Zhang et al., 2015). A previous study, involving many patients of different groups, including normozoospermia, oligoasthenoteratozoospermia, asthenozoospermia, and varicocoele, has shown the complete fatty acids lipidome as potential candidate markers of semen quality (Zerbinati et al., 2016). Further metabonomic analysis of fatty acids in seminal plasma between healthy and asthenozoospermic subjects based on gas chromatography/MS analysis showed high levels of oleic and palmitic acid in samples from infertile men (Tang et al., 2017).

The purpose of this study is an investigation of the lipid composition of human semen from patients with severe oligoasthenozoospermia in order to better elucidate the role of lipids on male fertility.

The analytical approach followed in the present paper consists of a combination of thin-layer chromatography (TLC) and MALDI-TOF/MS analyses not yet used for the study of lipids in oligoasthenozoospermia.

## Materials and Methods

### Materials

The matrix used for MALDI-TOF/MS analyses was 9-aminoacridine hemihydrate (9-AA) and was purchased from Acros Organics (Morris Plains, NJ, United States). All organic solvents used in extraction and MS analyses were commercially distilled, of the highest available purity, and purchased from Sigma-Aldrich, J.T. Baker, or Carlo Erba. The following commercial glycerophospholipids (used as standards): 1,2-dimyristoyl-*sn*-glycero-3-phosphate, 1,2-dimyristoyl-*sn*-glycero- 3-phospho-(1′-rac-glycerol), 1,2-dimyristoyl-*sn*-glycero-3-phos- pho-L-serine, 1,2-diphytanoyl-*sn*-glycero-3-phosphoethanola- mine, 1′,3′-bis[1,2-dimyristoyl-*sn*-glycero-3-phospho]-*sn*-glyce- rol, 1′,3′-bis[1,2-dioleoyl-*sn*-glycero-3-phospho]-*sn*-glycerol, were purchased from Avanti Polar Lipids, Inc. (Alabaster, AL, United States).

### Semen Samples and Processing

All aspects of the study were compliant with the Declaration of Helsinki. Semen samples from 33 couples undergoing ICSI treatment for male factor or other clinical indications at the Centre for Medically Assisted Procreation, Santa Maria Hospital of Bari, Italy were collected. All patients signed an informed consent for use of their biological material for scientific purpose. The ICSI option for the couples with normozoospermia was chosen when they required the oocyte cryopreservation. Only semen for ICSI cases were considered as less than 2 μl of the samples were used for the treatment itself contrary to the IVF cases where whole sample is needed for the treatment. Semen samples were collected by masturbation into a sterile container and, after the liquefaction, semen parameters were evaluated according to the World Health Organization guidelines (World Health Organization [WHO], 2010). The motility of each spermatozoon is graded as follows: PR (progressive motility): spermatozoa moving actively, either linearly or in a large circle, regardless of speed; NP (non-progressive motility): all other patterns of motility with absence of progression; IM (immotile spermatozoa): no movement.

Of 33 samples initially collected, only 20 were included in this study and the remaining 13 excluded in order to stress the differences in semen characteristics in terms of sperm number and/or motility. Of 20, 9 were normozoospermia (group A; control) and 11 were severe oligoasthenozoospermia (group B). The total progressively motile sperm cells/ejaculate were 157.4 ± 44.3 millions and 5.6 ± 6.1 millions (as mean ± SD) in groups A and B, respectively. After the collection, samples were frozen, and stored at −80°C until processed.

### Lipid Extraction

The whole semen samples (1 ml) (*S*) were centrifuged (800 *g* for 15 min) for separation of the spermatozoa (pellet) (*Sz*) from the seminal plasma (supernatant) (*Sp*). After collecting the supernatant, the separated spermatozoa pellet was washed once with 1 ml of phosphate-buffered saline, recollected and finally resuspended in 800 μl of distilled water. Total lipids were extracted from *Sz* and *Sp* obtained from all samples, using the procedure according to Bligh and Dyer method (methanol/chloroform/water; 2:1:0.8, by volume) (Bligh and Dyer, 1959). The lipid extracts were dried under N_2_ before weighing, and then dissolved in chloroform (final concentration 10 mg/ml). Alternatively, total lipids were extracted from 800 μl of whole semen (*S*), as above described.

### TLC Analyses

Total lipid extracts were analyzed by TLC on silica gel 60A plates (Merck, 20 × 10 cm, layer thickness 0.2 mm). The plates were washed twice with chloroform/methanol (1:1, by volume) and activated at 180°C before use. Total lipids were eluted with Solvent A (chloroform/methanol/acetic acid/water 85:15:10:3.5, by volume).

Lipid detection was carried out exposing the TLC plate to iodine vapor, for staining all classes of lipids. Moreover, the Azure A staining were performed in order to identify the sulfur-containing lipids in the TLC bands (Kates, 1986).

In order to analyze the various components of the lipid extracts in detail, bands present on plates were scraped and lipids extracted from silica were then analyzed by negative and positive ion modes MALDI-TOF/MS.

Briefly, the polar lipid components of the total lipid extracts of semen samples were separated by TLC in Solvent A. Lipids were visualized by staining with iodine vapor and then were eluted and recovered from the scraped silica, as previously described (Lobasso et al., 2012; Lopalco et al., 2017). Isolated and purified phospholipids were dissolved in chloroform (1 mg/ml).

### MALDI-TOF Mass Spectrometry

MALDI-TOF mass spectra of lipid extracts were generally acquired on a Bruker Microflex LRF mass spectrometer (Bruker Daltonics, Bremen, Germany). The system utilizes a pulsed nitrogen laser, emitting at 337 nm, the extraction voltage was 20 kV and gated matrix suppression was applied to prevent detector saturation. For each mass spectrum, 2000 single laser shots (sum of 4 × 500) were averaged. The laser fluence was kept about 5% above threshold to have a good signal-to-noise ratio. All spectra were acquired in reflector mode (detection range: 200–2000 mass/charge, *m/z*) using the delayed pulsed extraction; spectra were acquired in negative and positive ion modes. Spectral mass resolutions and signal-to-noise ratios were determined by the software for the instrument “Flex Analysis 3.3” (Bruker Daltonics).

A mix containing: 1,2-dimyristoyl-*sn*-glycero-3-phosphate, 1,2-dimyristoyl-*sn*-glycero-3-phospho-(1′-rac-glycerol), 1,2-di- myristoyl-*sn*-glycero-3-phospho-L-serine, 1,2-diphytanoyl-*sn*- glycero-3-phosphoethanolamine, 1′,3′-bis[1,2-dimyristoyl-*sn*-gl- ycero-3-phospho]-*sn*-glycerol, 1′,3′-bis[1,2-dioleoyl-*sn*-glycero-3-phospho]-*sn*-glycerol, was always spotted next to the sample as external standard and an external calibration was performed before each measurement in negative ion mode; the mass range of the authentic standards is 590–1450 atomic mass units (*amu*). A mix containing 1,2-distearoyl-sn-glycero-3-phosphocholine, 1,2-dimyristoyl-sn-glycero-3-phosphocholine, 1,2-di-O-phytanyl-sn-glycero-3-phosphocholine was always spotted next to the sample as external standard and an external calibration was performed before each measurement in positive ion mode.

Post Source Decay (PSD) spectra were acquired on a Bruker Microflex mass spectrometer (Bruker Daltonics, Bremen, Germany), as previously described (Fuchs et al., 2007). Briefly, the precursor ions were isolated using a time ion selector. The fragment ions were refocused onto the detector by stepping the voltage applied to the reflectron in appropriate increments. This was done automatically by using the “FAST” (fragment analysis and structural TOF) subroutine of the Flex Analysis software. Mass accuracy of our instrument is 200 ppm (external calibration). A specific lipid database (Lipid Maps Database)^1^ (LIPID MAPS Lipidomics Gateway) was used to facilitate and confirm the assignment of lipid species.

The signals of the MALDI mass spectra (three replicates/sample) were exported in a final matrix in a compatible format for the multivariate analysis with ClinProTools3.0 software and *t*-test was used to confirm significant differences between the two lipid patterns. A *p*-value from Student’s *t*-test <0.05 was set as the threshold to define significant differences between the peaks present in the two series of spectra.

### Preparation of Samples for MALDI-TOF/MS Lipid Analyses

A total of 3 μl lipid extract in chloroform solution (10 mg/ml) was diluted in 27 μl of 2-propanol/acetonitrile (60/40, by volume), then 10 μl of the diluted solution were mixed with 10 μl of matrix solution (9-AA, 10 mg/ml in 2-propanol/acetonitrile 60/40, by volume), as previously described (Sun et al., 2008; Angelini et al., 2015). The resulting lipids-matrix solution was then spotted onto the MALDI target (Micro Scout Plate, MSP 96 ground steel target) in droplets of 0.35 μl and analyzed as described above.

After the evaporation of the matrix solvent, the samples are ready to be directly analyzed with MALDI-TOF/MS.

## Results

### Lipid Analysis of Semen, Spermatozoa and Seminal Plasma by TLC and MALDI-TOF/MS

**FIGURE 1:**
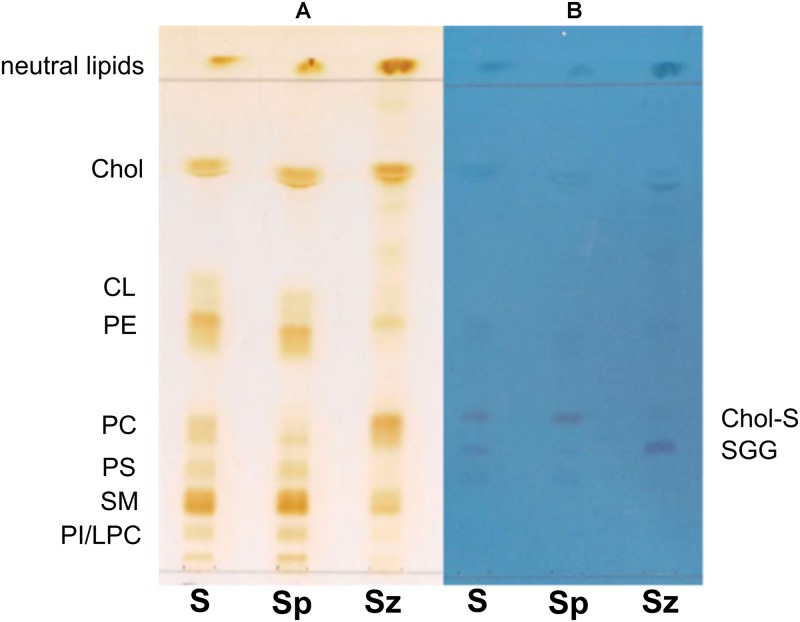
Comparison of TLC lipid profiles of semen, seminal plasma and spermatozoa. Total lipids were extracted from whole semen (*S*) as well as from related spermatozoa (*Sz*) and seminal plasma (*Sp*) obtained from a man with normozoospermia and analyzed by TLC with the same solvent, as described in Methods. All species of lipids are stained by iodine vapor **(A)**; sulfur-containing lipids are stained by azure A reagent **(B)**. Lipid bands are indicated by their abbreviations: phosphatidylinositol (PI), lysophosphatidylcholine (LPC), sphingomyelin (SM), phosphatidylserine (PS), phosphatidylcholine (PC), phosphatidylethanolamine (PE), cardiolipin (CL), cholesterol (Chol), cholesterol sulfate (Chol-S), and seminolipid (SGG).

Total lipids were extracted from whole semen (*S*) as well as from related spermatozoa (*Sz*) and seminal plasma (*Sp*) obtained from a man with normozoospermia; then the total lipids were analyzed by TLC and MALDI-TOF/MS. Figure 1 shows the comparison between the representative TLC lipid profiles of the three samples. The use of an acid eluent allows the separation of polar lipids along the TLC plate, while neutral lipids run close to the solvent front. Lipid bands are stained by iodine vapors (Figure 1A) and azure A (Figure 1B); the first staining shows all species of lipids on TLC plate, whereas the second one is specific for sulfur-containing lipids only. Individual polar lipids in Figure 1 were identified by comparison of their retention factor (*R*_*f*_) values with those of lipid standards (not shown). The polar lipids of semen were identified (in *R*_*f*_ order) as phosphatidylinositol (PI)/lysophosphatidylcholine (LPC), sphingomyelin (SM), phosphatidylserine (PS), phosphatidylcholine (PC), phosphatidylethanolamine (PE), cardiolipin (CL), and cholesterol (Chol). Individual bands of each TLC lipid profile have been isolated and MALDI-TOF/MS analyses (in either negative or positive ion mode) of the lipids extracted from silica confirmed above assignments (not shown).

It can be observed that Chol, PE, and SM are the most abundant species in the semen lipid profile [see (*S*) profile in Figure 1A]. The *Sz* and *Sp* lipid profiles are qualitatively similar to the *S* profile, but it can be noted that the lipid band corresponding to PC is more abundant in spermatozoa than in seminal plasma. On the contrary, the other lipid bands, corresponding to CL, PE, PS, SM, and PI/LPC, are less abundant in cells than in the seminal plasma lipid profile.

The use of the staining for sulfur-containing lipids helped us to identify some overlapping lipids present in the middle of TLC (Figure 1B); in particular, two bands having *R*_*f*_ next to PC have been attributed to (in *R*_*f*_ order) seminolipid, also known as SGG, and Chol-S, a derivative of Chol. Both lipids are visible in the three lipid profiles. The Chol-S is more abundant in the lipid profile of seminal plasma, whereas SGG in that of spermatozoa, as previously described (Schiller et al., 2000; Lessig et al., 2004; Engel et al., 2017).

To gain further information on the lipid composition of the three samples, lipid extracts were analyzed by MALDI-TOF/MS, in negative and positive ion mode. According to the previous literature, we found lipid classes that comprise a number of molecular species of phospholipids and glycolipids due to different aliphatic acyl-, alkyl- or alkenyl-chains (Flesch and Gadella, 2000). The main signals detected in the MALDI-TOF mass spectra of the lipid extracts, attributable to the negative [M−H]^–^ and positive [M+H]^+^ molecular ions of lipid species, are collected in Table 1.

**TABLE 1:** Assignments of *m/z* values detected in MALDI-TOF mass spectra of lipids from semen.

***m/z* Value**	**Assignment [M–H]^–^**	**Assignment [M+H]^+^**
465.3	Chol-S	
496.6		LPC (16:0)
687.7	SM (16:0)	
701.6	SM (16:0)	
703.7		SM (16:0)
716.6	PE (34:1)	
725.6		SM (16:0) + Na^+^
726.7	PE (P−36:2)	
728.7	PE (P−36:1)	
732.7		PC (32:1)
734.6		PC (32:0)
742.7	PE (36:2)	
744.6	PE (36:1)	
746.7	PS (P−34:0)	
748.7	PS (O−34:0)	
750.7	PE (O−38:5)	
758.7		PC (34:2)
760.7		PC (34:1)
767.6	SGG (O−30:0)	
782.8		PC (36:4)/PC (34:1) + Na^+^
784.8		PC (36:3)
786.8		PC (36:2)
788.7	PS (36:1)	PC (36:1)
792.7		PC (O−38:6)
795.7	SGG (O−32:0)	
806.7		PC (38:6)
809.7	SGG (O−33:0)/PI (32:0)	
821.7	SGG (O−34:1)/SGG (P−34:0) PI (O−34:1)/PI (P−34:0)	
823.7	SGG (O−34:0)/PI (O−34:0)	
834.9		PC (40:6)
835.8	PI (34:1)	
861.8	PI (36:2)	
863.8	PI (36:1)	
883.8	PI (38:5)	
885.8	PI (38:4)	
887.8	PI (38:3)	
909.8	PI (40:6)	
1283.7		NG
1352.3	CL (64:0)	
1409.7		NG
1466.1		NG
1494.5		NG
1577.1		NG
1603.2		NG

*The numbers (x:y) denote the total length (as carbon numbers) and number of double bonds of acyl chains respectively. O-x:y = alkyl-acyl chains; P-x:y = alkenyl-acyl chains; NG = neutral glycosphingolipid.*

**FIGURE 2:**
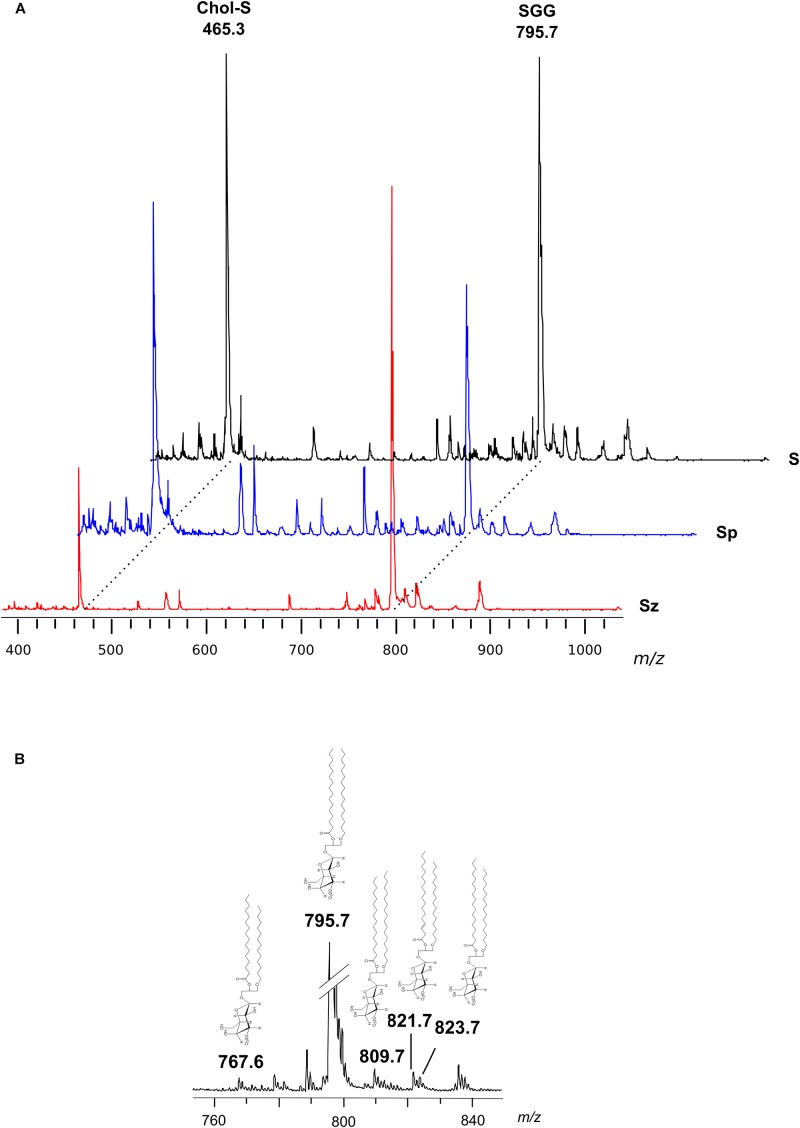
Negative ion mode MALDI-TOF/MS analyses of the lipid extracts of semen, seminal plasma and spermatozoa. Total lipids were extracted from whole semen (*S*) as well as from related spermatozoa (*Sz*) and seminal plasma (*Sp*) obtained from a man with normozoospermia. In panel **(A)**, the typical lipid profiles are shown. The main peaks are attributable to Chol-S [M–H]^–^ at *m/z* 465.3 and SGG O-32:0 [M–H]^–^ at *m/z* 795.7. In panel **(B)**, the enlargement of the region at *m/z* 760–840 of mass spectrum of *S* is shown. The peaks at *m/z* 767.6, 809.7, 821.7, and 823.7 are assigned to other SGG species (O-30:0, O-33:0, O-34:1 or P-34:0, and O-34:0, respectively); their molecular structures are also drawn.

Figure 2A shows the comparison between the representative lipid profile of *S*, *Sz*, and *Sp*, obtained by negative ion MALDI-TOF/MS analysis of the lipid extracts. All the lipid patterns are dominated by two main signals at *m/z* 465.3 and *m/z* 795.7, attributable to Chol-S and SGG, respectively. The intensity of both the peaks is very higher than the other ones in the spectra. In fact it is known that the sensitivity of MALDI-TOF technique is particularly high for sulfated glycolipids and sulfatides, which exhibit an extremely high tendency to ionize in the presence of matrix 9-AA (Angelini et al., 2010; Cheng et al., 2010). By comparing the intensity of the main MALDI signals in the mass spectra of *Sp* and *Sz*, it can be clearly observed that the peak at *m/z* 465.3 has higher intensity in the first profile, while the peak at *m/z* 795.7 has higher intensity in the second one, according to previous TLC data (Figure 1).

In particular, the SGG at *m/z* 795.7 consists of a sulfogalactosylglycerol with a *sn*-1 alkyl chain C16:0 and *sn*-2 acyl chain C16:0, i.e., SGG O-32:0. The chemical structure of SGG O-32:0 was further validated by PSD analysis (see Supplementary Figure S1). Other SGG species are also detected in the mass spectra, as minor MALDI signals, at *m/z* 767.6, 809.7, 821.7, and 823.7 corresponding to seminolipid having different alkyl- and alkenyl- chains in the molecular structure (O-30:0, O-33:0, O-34:1 or P-34:0, and O-34:0, respectively) (see Figure 2B), previously described (Goto-Inoue et al., 2009).

Minor MALDI signals detected in the mass spectra showed in Figure 2A were assigned to various species of the phospholipids PI, SM, PS, and PE (see Table 1). It is noteworthy that among the minor signals of spermatozoa lipid profile, a small peak at *m/z* 1352.3 has been detected and assigned to the dimeric phospholipid CL 64:0, i.e., having four palmitic acids in its molecular structure, according to a recent study (Ren et al., 2019).

The lipid extracts of *S*, *Sz*, and *Sp*, have been analyzed by positive ion mode MALDI-TOF/MS (see Supplementary Figure S2); MALDI peaks attributable to various species of the phospholipids PC and SM and to the glycosphingolipids NG were collected in Table 1. By comparing the lipid patterns, it can be observed that the intensities and the number of PC signals are more complex in the spermatozoa mass spectra than those in the seminal plasma, suggesting a higher PC content, in agreement with TLC data above shown in Figure 1A. Furthermore, a LPC species has been detected at *m/z* 496.6, assigned to LPC 16:0, as previously described in Schiller et al. (2000), Lessig et al. (2004). In agreement with above TLC results, the peak assigned to LPC was higher in the lipid profile of *Sp* than in that of *Sz*.

### Lipid Profiling of Semen From Patients With Severe Oligoasthenozoospermia

We investigated the possible change of levels of some semen lipids between subjects with normozoospermia (group A) and patients with severe oligoasthenozoospermia (group B). The values of semen parameters from men belonging to the two groups are reported in Table 2.

**TABLE 2:** Values of semen parameters from men with normozoospermia (group A) and men with oligoasthenozoospermia (group B).

**Group**	**Case N.**	**Semen volume (ml)**	**Sperm concentration (millions/ml)**	**TSN (millions/ejaculate)**	**IM (millions/ml)**	**NP (millions/ml)**	**PR (millions/ml)**	**PR (%)**	**Total PR (millions)**
A	#66	3.7	49.0	181.3	18.0	1.0	30.0	61%	111.0
	#76	3.0	92.0	276.0	30.0	10.0	52.0	57%	156.0
	#97	3.2	75.0	240.0	32.0	1.0	42.0	56%	134.4
	#07	3.0	81.0	243.0	25.0	2.0	54.0	67%	162.0
	#09	2.2	135.0	297.0	70.0	10.0	55.0	41%	121.0
	#24	3.9	68.0	265.2	32.0	0	36.0	53%	140.4
	#53	3.7	87.0	321.9	15.0	2.0	70.0	80%	259.0
	#61	2.9	66.0	197.2	15.0	3.0	50.0	74%	145.0
	#63	2.5	140.0	350.0	65.0	0.0	75.0	54%	187.5
B	#70	1.9	7.6	14.4	3.7	0.4	3.5	46%	6.65
	#72	2.2	0.8	1.8	0.5	0	0.3	38%	0.66
	#74	1.3	22.0	28.6	12.0	0.8	9.2	42%	11.96
	#77	3.4	0.006	0.02	0.004	0.0004	0.0012	21%	0.004
	#42	2.2	0.026	0.06	0.02	0	0.006	23%	0.013
	#83	1.5	15.0	22.5	6.0	1.0	8.0	53%	12.0
	#84	4.0	3.8	15.2	3.5	0.2	0.1	3%	0.40
	#86	1.1	26.3	28.9	25.0	0.3	1.0	4%	1.10
	#88	3.0	6.3	18.9	4.0	2.0	0.3	5%	0.90
	#89	3.0	18.0	54.0	14.0	0.5	3.5	19%	10.50
	#93	1.7	11.2	19.0	2.9	0.5	7.8	70%	13.26

*Semen were evaluated according to World Health Organization [WHO] (2010) with Makler chamber. TSN, total sperm number; IM, immotile sperm cells; NP, non-progressive motile sperm cells; PR, progressive motile sperm cells; Total PR, total progressive motile sperm cells.*

As above described in section “Materials and Methods,” total lipids were extracted from an aliquot of each semen sample. The contents (mean total ± SD) of total lipids in samples of the group A and B were 1.63 ± 0.42 and 1.33 ± 0.30 mg/ml, respectively.

**FIGURE 3:**
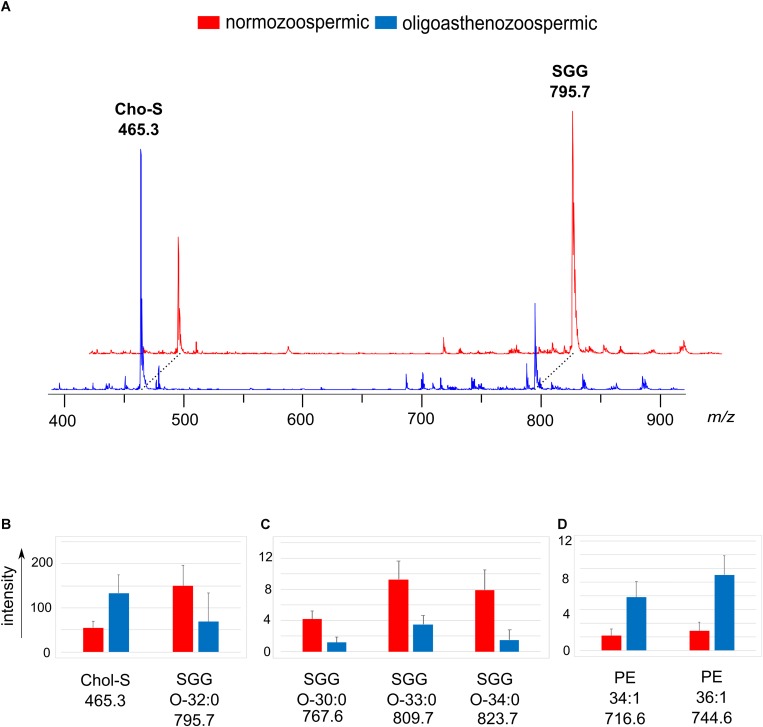
Negative ion mode MALDI-TOF/MS analyses of the lipid extracts of semen of normozoospermic subjects and oligoasthenozoospermic patients. In panel **(A)**, two typical lipid profiles are shown. The histograms show the significant differences in intensity of lipid peaks at *m/z* 465.3 and 795.7 corresponding to Chol-S and SGG O-32:0 **(B)**, at *m/z* 767.6, 809.7 and 823.7 corresponding to main SGG species **(C)**, at *m/z* 716.6, 744.6 and 778.7 corresponding to PE species **(D)**. A *p*-value from Student’s *t*-test <0.05 was set as the threshold to define significant differences between the peaks present in the two series of spectra. Data are reported as the average value ± SD.

Figure 3A shows the comparison between the representative lipid profiles of normozoospermic and oligoasthenozoospermic samples, obtained by negative ion MALDI-TOF/MS analysis of the semen total lipid extracts. By comparing series of replicates of normozoospermic and oligoasthenozoospermia semen mass spectra, we found significant differences in intensity of some lipid peaks (Figures 3B–D).

The most evident change in the MALDI signals is relative to the main peaks in the mass spectra, previously assigned to two sulfur-containing lipids. The peak at *m/z* 795.7 attributable to SGG O-32:0 was significantly lower, whereas the peak at *m/z* 465.3 attributable to Chol-S was higher in oligoasthenozoospermic sample (see mass spectra in Figure 3A).

The mean intensity of the Chol-S and SGG O-32:0 signals were 55.1 ± 14.0 and 150.0 ± 47.6 in normozoospermic samples and 133.9 ± 41.0 and 68.6 ± 65.3 in oligoasthenozoospermic patients, respectively (see histograms in Figure 3B).

A significant decrease of the intensity of other peaks at *m/z* 767.6, 809.7 and 823.7 assigned to other SGG species (O-30:0, O-33:0 and O-34:0, respectively) in mass spectra of oligoasthenozoospermic samples was also found (see histograms of Figure 3C). We found other significant differences in intensities of lipid peaks between the two groups of mass spectra: the signals at *m/z* 716.6 and *m/z* 744.6 assigned to PE species (34:1 and 36:1, respectively) significantly increased in lipid mass spectra of oligoasthenozoospermic samples (see histograms of Figure 3D).

**FIGURE 4:**
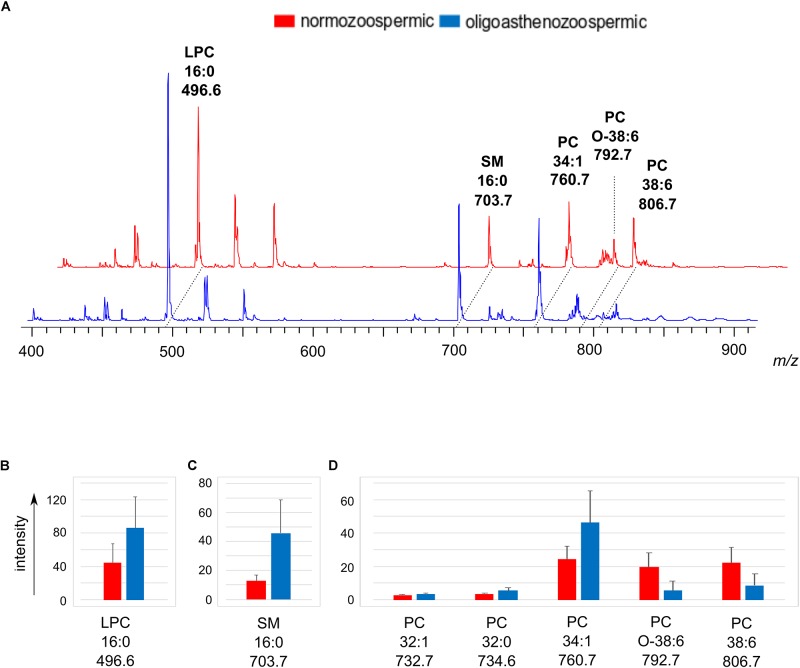
Positive ion mode MALDI-TOF/MS analyses of the lipid extracts of semen of normozoospermic subjects and oligoasthenozoospermic patients. In panel **(A)**, two typical lipid profiles are shown. Main species of lipid classes are reported: LPC 16:0 [M+H]^+^ at m/z 496.6; SM 16:0 [M+H]^+^ at m/z 703.7; PC 34:1 [M+H]^+^ at m/z 760.7; PC O-38:6 [M+H]^+^ at m/z 792.7 and PC 38:6 [M+H]^+^ at m/z 806.7. The histograms show the significant differences in intensity of lipid peaks at *m/z* 496.6 corresponding to LPC **(B)**, at *m/z* 703.7 corresponding to SM **(C)**, at *m/z* 732.7, 734.6, 760.7, 792.7, 806.7 corresponding to PC species **(D)**. A *p*-value from Student’s *t*-test <0.05 was set as the threshold to define significant differences between the peaks present in the two series of spectra. Data are reported as the average value ± SD.

The lipid extracts of semen from normozoospermic and oligoasthenozoospermic subjects were also analyzed by positive ion mode MALDI-TOF/MS to gain further information on the lipid changes of the two groups of samples. Figure 4A shows the comparison between the representative lipid profiles of normozoospermic and oligoasthenozoospermic samples and the significant differences in intensity of some lipid peaks are illustrated in the Figures 4B–D. Both the mass spectra contain signals assigned to PC, LPC, and SM species as main peaks.

By comparing the mass spectra, we found that the signal at *m/z* 703.7, corresponding to the molecular ion [M+H]^+^ of SM 16:0, significantly increases in the oligoasthenozoospermic lipid profiles (see Figure 4C).

As regards the PC species, the statistical analysis showed that the MALDI signals assigned to species having saturated (PC 32:0 at *m/z* 734.6) or monounsaturated fatty acids chains (PC 32:1 and PC 34:1, at *m/z* 732.7 and 760.7, respectively) increased. Whereas PC species with polyunsaturated chains (PC O-38:6 and PC 38:6, at *m/z* 792.8 and *m/z* 806.7, respectively) decreased in the oligoasthenozoospermic samples (see Figure 4D). Finally, the peak at *m/z* 496.6, assigned to the PC derivative LPC 16:0, was relatively high in both the spectra, but significantly increased in oligoasthenozoospermic samples (see Figure 4B). We suggest that the significant higher saturated LPC content may be derived from the abundant PC 38:6 due to the loss of the oxidatively modified unsaturated residue, according to Nimptsch et al. (2014).

**FIGURE 5:**
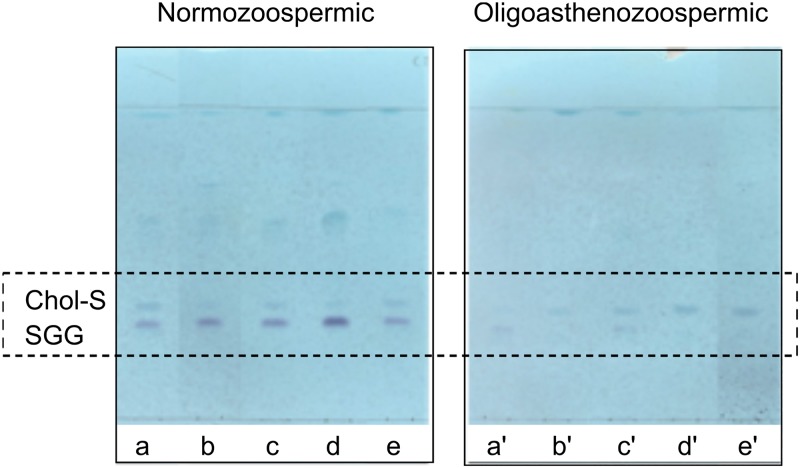
Comparison of TLC sulfolipid profiles of semen of normozoospermic subjects and oligoasthenozoospermic patients. Total lipids were extracted from semen samples of normozoospermic (a, b, c, d, e) and oligoasthenozoospermic (a′, b′, c′, d′, e′) subjects and then eluted with the same solvent as in TLC showed in Figure 1. Azure A reagent was used to stain sulfur-containing lipids only. Lipid bands assigned to cholesterol sulfate (Chol-S) and seminolipid (SGG) are indicated by their abbreviations.

The lipid changes observed in the MALDI lipid profiles of semen from patients with oligoasthenozoospermia and subjects with normozoospermia have been confirmed by TLC analysis. The comparison of representative TLC polar lipid profiles belonging to the two groups of semen samples has been shown in the Supplementary Figure S3. Finally, Figure 5 shows very clearly the differences in the content of the two sulfolipids, i.e., SGG and Chol-S, when lipid profiles of semen from normozoospermic subjects and oligoasthenospermia patients are compared by TLC analysis.

## Discussion

The interaction between oocyte and sperm and the subsequent fertilization are highly regulated processes. Capacitation (i.e., the activation of sperm cells in the female genital tract) and the acrosome reaction (i.e., an exocytotic process of hydrolytic enzymes required form sperm penetration) are two specific events that are required for fertilization. Important changes in the plasma membrane of the sperm cell are involved in these processes.

In the past, several analytical techniques have been used to study the lipid composition of spermatozoa and seminal plasma during fertilization and the sperm maturation. Nowadays the MALDI-TOF/MS technology is considered a rapid and easy tool to have novel information on lipid molecular species and components of biological samples. In the present study, we have used MALDI-TOF/MS (in both negative and positive ion mode) together with TLC to elucidate the lipid composition of whole semen as well as of its components, spermatozoa and seminal plasma.

PC, PE, and SM are the most abundant phospholipids we found in spermatozoa, as previously described (Evans et al., 1980; Mann and Lutwak-Mann, 1981; Schiller et al., 2000; Lessig et al., 2004). As regards CL, we found small amounts in the spermatozoa TLC lipid profile, but it is noteworthy that the only CL species detected by MALDI-TOF/MS, at *m/z* 1352.3, is a tetrapalmitoyl-CL (CL 64:0). In general, unsaturated CL species are described in mitochondrial membrane and the importance of unsaturated fatty acids for CL function has been described (Ren et al., 2014; Lu and Claypool, 2015). The fully saturated CL 64:0 species was first discovered in testicular germ cells of rat (Wang et al., 2007) and recently found also in the testes of human (Ren et al., 2019). It is noteworthy that this CL is an extra-mitochondrial CL, being localized in mitochondria-derived membranes assembled into the acrosome (Ren et al., 2019).

Furthermore, by comparing the lipid extracts of spermatozoa and seminal plasma by TLC, we showed that the lipid profile of seminal plasma has higher levels of the phospholipids PS, PE, and the lyso-compound LPC than the spermatozoa. As regards sulfur-containing lipids, various species of seminolipid SGG having different acyl-, alkyl- or alkenyl-chains have been detected by MALDI both in spermatozoa and in seminal plasma. SGG species are more abundant in the spermatozoa membrane than in seminal plasma, as previously described (Schiller et al., 2000; Lessig et al., 2004; Engel et al., 2017). Chol-S represents about 6% of total Chol both in spermatozoa and seminal plasma (Sion et al., 2001).

It is well-known that the fluidity of the sperm membrane is very high due to the unusually high proportion of long-chain PUFAs and that the fluidity and the flexibility of the membrane are very important for the development of sperm motility (Lenzi, 1996; Ladha, 1998).

The aim of this study was to identify possible sperm lipid changes and molecular markers as potential biomarkers for the reduction of motility.

As regards the phospholipids present in the lipid profiles of patients here analyzed, we observed an increase of content of species containing saturated and monounsaturated fatty acids and in parallel a decrease of species containing PUFAs. As the high degree of fatty acid unsaturation is a physiological characteristic of sperm membrane lipids and crucial for the fluidity of the membrane, our data might correlate with the reduction of sperm motility in asthenozoospermia and highlight the role of oxidative stress in its mechanisms.

As regards the main sulfated lipids, the MALDI and TLC comparative analyses of lipid profiles of semen from the two groups of subjects here analyzed suggest an alteration in their levels. Statistical analyses of mass spectra showed that the Chol-S and SGG contents were significantly different between the two groups. In particular the Chol-S/SGG ratio increases about five times (i.e., Chol-S/SGG changes from about 0.4 in samples from normozoospermic subjects to about 2 in those from oligoasthenozoospermic patients).

According the World Organization Guidelines (World Health Organization [WHO], 2010), the majority of the samples (8/9) of the group A exceed the 50th percentile either for the %PR (55%) or for the Total PR (140.25) whereas most samples (9/11) of the group B are below the 2.5th percentile. As shown in Table 2, the total progressive motility (Total PR, Table 2) of group A ranges from 111.0 to 259.0 (mean value 157.4 ± 44.3 millions), while that of group B ranges from 0.004 to 13.26 (mean value 5.57 ± 6.10 millions). It can be seen that the total sperm number (TSN, Table 2) of the group A ranges from 181.3 to 350.0 millions of spermatozoa, while that of the group B is much lower (from 0.02 to 54.0 millions of spermatozoa).

As the seminolipid SGG is more abundant in spermatozoa membrane than in seminal plasma, it can be considered that the significant decrease of SGG content found in semen of patients with severe oligoasthenozoospermia may be correlated with the low sperm concentration in these samples compared to samples from normozoospermic subjects. It can be interesting, in the future, to compare the SGG content between subjects with normal cell number and normal % PR and those with normal number and low % PR.

In any case, to our knowledge, this is the first report about the determination of Chol-S/SGG ratio by MALDI-TOF/MS in human semen of patients with oligoasthenozoospermia. Our findings suggest that the lipid ratio can be considered as lipid biomarker for semen quality in the clinical practice. Although the role of the altered lipid ratio in fertility is difficult to determine as four women in each group became pregnant following the treatment (mean female age was 41.4 ± 2.9 and 36.0 ± 3.3 (as mean ± SD) in the groups A and B, respectively).

These results are very preliminary and it is difficult to find any association between lipid content and single specific sperm characteristics, such as motility, total motile sperm and concentration. Furthermore, it will be interesting to investigate any association to other data regarding subjects (such as age, BMI, smoking, comorbidities, etc.).

It is known that an important process of membrane lipid change occurring during the epididymal transit and the capacitation process is the removal of Chol and other sterols from the sperm surface; it is known that it creates a more fluid sperm membrane necessary for the acrosome reaction and penetration of the egg cell (Harrison, 1996; Osheroff et al., 1999). The progressive reduction in Chol levels sustains the fertility potential of mature spermatozoa; on the other hand high levels of membrane Chol are expected to have an opposite effect (Needham and Evans, 1988). Our findings about the higher content of Chol-S in semen from oligoasthenozoospermic patients might parallel with a recent study in which Chol levels were dramatically higher in sperm membrane from infertile patients than from fertile men (Garolla et al., 2018). Furthermore higher levels of oxidized Chol derivatives, 7-beta-hydroxycholesterol, and 7-keto-cholesterol, have been recently described in infertile sperm from asthenozoospermic patients (Garolla et al., 2018). As SGG is exclusively present in the sperm, whereas Chol-S is also a component of blood (Drayer and Lieberman, 1965), it may be interesting to measure both the Chol and Chol-S also in the blood in order to verify patient specific differences. Also studies on isolated spermatozoa may clarify any role of the alteration of Chol-S/SGG ratio in sperm function.

Again, this is a preliminary study on limited number of subjects and further studies on larger sample number are needed to better define the relationship between the altered sulfated lipid ratio, the PUFA composition of the phospholipids and sperm cell functions.

## Data Availability Statement

The datasets generated for this study are available on request to the corresponding author.

## Ethics Statement

The study was approved by the Ethics Committee of the Azienda Ospedaliero-Universitaria Consorziale Policlinico of Bari. The patients/participants provided their written informed consent to participate in this study.

## Author Contributions

All authors listed made a substantial, direct and intellectual contribution to the work. They read and approved the final manuscript for publication.

## Conflict of Interest

The authors declare that the research was conducted in the absence of any commercial or financial relationships that could be construed as a potential conflict of interest.

## Footnotes

1 http://www.lipidmaps.org
