# Supplementary material for: Alteration of Cholesterol Sulfate/Seminolipid Ratio in Semen Lipid Profile of Men With Oligoasthenozoospermia
Source: Front Physiol. 2019 Oct 29;10:1344. doi: 10.3389/fphys.2019.01344 (PMC6828844; doi:10.3389/fphys.2019.01344)
Supplement: Supplementary file 1 [file Table_1.DOCX]

**Supplementary figures**


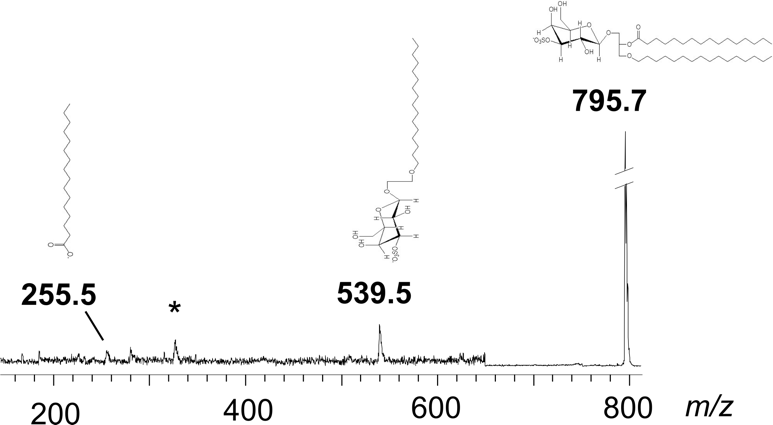


**Supplementary Figure 1.** PSD analysis of peak at *m/z* 795.7, corresponding to SGG O-32:0. Ion fragments correspond to SGG loss of sn-2 (*m/z* 539.5) and fatty acid 16:0 (sn-2 acyl chain) (*m/z* 255.5). Asterisk shows unidentified ion. The molecular structure and its fragments are reported close to the corresponding peaks.


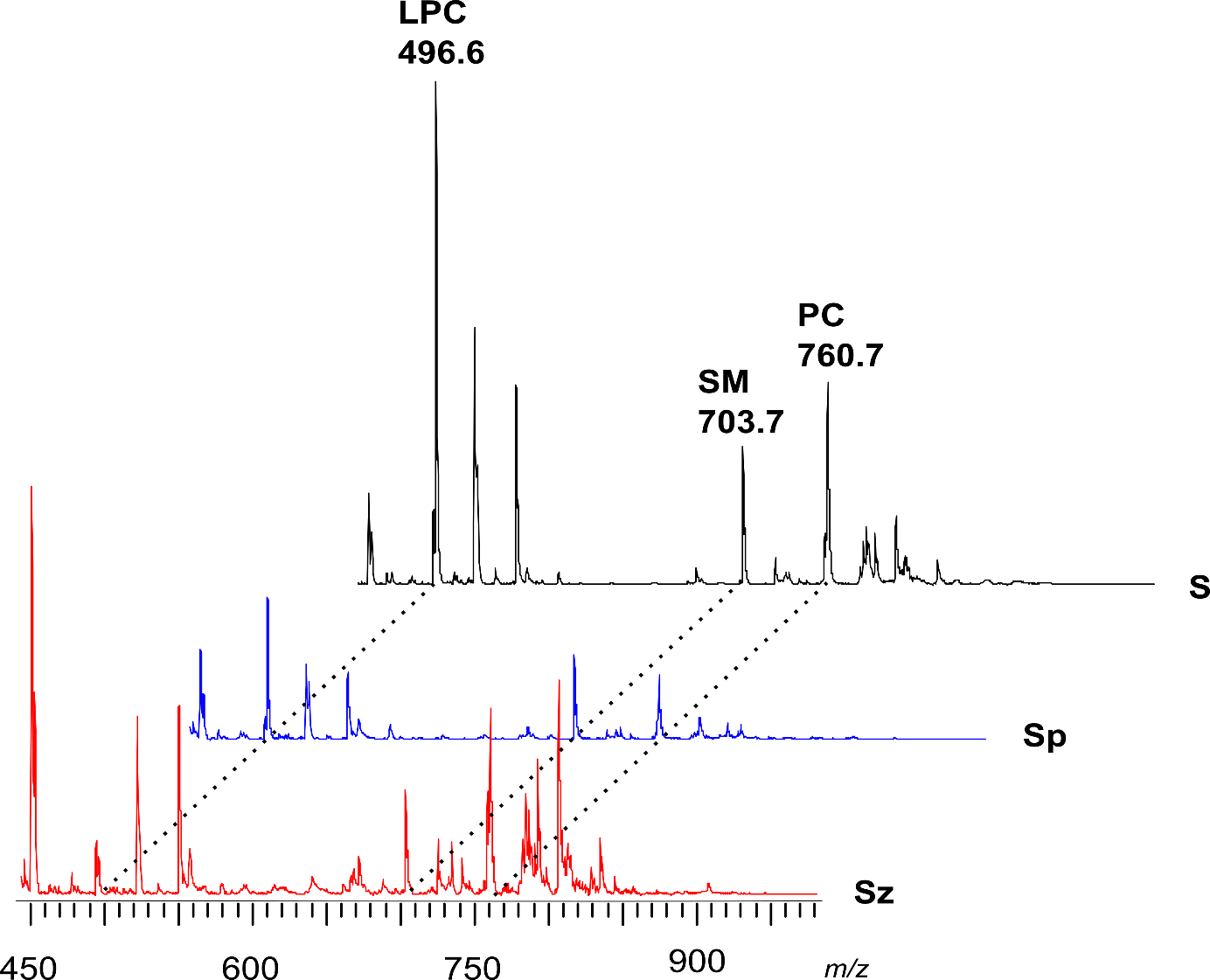


**Supplementary Figure 2.** Positive ion mode MALDI-TOF/MS analyses of the lipid extracts of sperm (S), seminal plasma (Sp) and spermatozoa (Sz). In the three mass spectra the main LPC (16:0), SM (16:0) and PC (34:1) species are reported at *m/z*: 496.6, 703.7 and 760.7, respectively.

~~
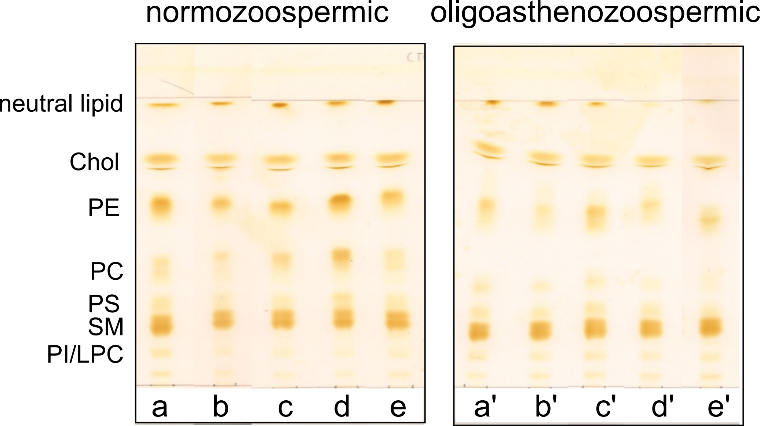
~~

**Supplementary Figure 3.** TLC lipid profiles of normozoospermic subjects and asthenozoospermic patients. The TLCs show the representative lipid profiles in five samples of normozoospermic (a, b, c, d, e) and asthenozoospermic (a_1_, b_1_, c_1_, d_1_, e_1_) sperm. All lipid species were stained by iodine vapors.
